# Long‐term stability of adjunctive use of enamel matrix protein derivative on porcine‐derived xenograft for the treatment of one‐wall intrabony defects: A 4‐year extended follow‐up of a randomized controlled trial

**DOI:** 10.1002/JPER.21-0254

**Published:** 2021-06-09

**Authors:** Jae‐Hong Lee, Seong‐Nyum Jeong

**Affiliations:** ^1^ Department of Periodontology Daejeon Dental Hospital, Institute of Wonkwang Dental Research, Wonkwang University College of Dentistry Daejeon Korea

**Keywords:** follow‐up studies, periodontal diseases, randomized controlled trial, surgical flaps

## Abstract

**Background:**

The long‐term outcomes of demineralized porcine bone matrix (DPBM) in combination with enamel matrix protein derivative (EMD) for the treatment of one‐wall intrabony defects have not yet been evaluated. Therefore, this study aimed to assess the clinical, radiographic, and patient‐reported outcomes of regenerative therapy using DPBM with EMD (test group) in comparison with DPBM alone (control group) for the treatment of one‐wall intrabony defects in the molar regions.

**Methods:**

Thirty‐four patients (control group, *n* = 18, and test group, *n* = 16) were available at the 4‐year follow‐up assessment. Clinical (probing pocket depth and clinical attachment level [CAL]), radiographic (defect depth and width), and patient‐reported (Oral Health Impact Profile [OHIP]‐14) parameters were evaluated at baseline, 2 years, and 4 years after regenerative treatment.

**Results:**

Both treatment modalities, with and without adjunctive use of EMD, resulted in significant improvement of clinical (mean gain in CAL of 1.58 ± 1.34 mm), radiographic (mean defect width fill of 2.41 ± 0.90 mm), and oral health‐related quality of life outcomes at 2 years after regenerative treatment of one‐wall intrabony defects (*P* < 0.001), which has been sustained over a 4‐year follow‐up period. Particularly, OHIP‐14 scores revealed a statistically significant reduction in physical pain, psychological discomfort, and physical disability (*P* < 0.05).

**Conclusions:**

The clinical, radiographic, and patient‐reported outcomes were significantly improved when DPBM was used in the regenerative treatment, but no additional benefits were observed with the adjunctive use of EMD.

## 1 INTRODUCTION

Periodontal regenerative therapy is a predictable and effective treatment modality for the formation of new alveolar bone, cementum, and periodontal ligament on a previously diseased root surface.^1^ In particular, guided tissue regeneration (GTR) has shown reliable and convincing clinical and functional outcomes in the past decades, with a long‐term survival and success rate.^2^ One systematic review reported that the survival rate of GTR ranged from 83.3% to 100% over 5 years, and another consensus report from the American Academy of Periodontology regeneration workshop also confirmed that clinical and radiographic improvements in periodontal condition could be maintained over a period of up to 10 years.^2^, ^3^

Enamel matrix derivative (EMD) has been widely used in periodontal regenerative treatment, especially for contained intrabony periodontal defects.^4^, ^5^ A recent systematic review and meta‐analysis of 13 randomized controlled clinical trials (RCTs) indicated that the use of EMD was associated with significant adjunctive benefit of clinical attachment level (CAL) gain (1.34 mm, 95% confidence interval [CI] 0.95–1.73) for the treatment of deep intra‐bony defects >3 mm, compared with open flap debridement alone.^4^ Among the bone grafting biomaterials that are successfully used as scaffolds with EMD, in particular, deproteinized bovine bone mineral (DBBM) shows an additional improvement in clinical outcomes when combined with EMD and is considered effective especially in large or non‐contained intrabony defects.^4^, ^5^

Demineralized porcine bone matrix (DPBM) is one of the successfully used xenografts in various periodontal and implant surgeries including guided bone regeneration, alveolar ridge preservation, and sinus augmentation and provides stable and reliable results clinically and radiographically.^6^, ^7^, ^8^, ^9^ In this previous RCT, we demonstrated that DPBM with and without the adjunctive use of EMD showed favorable biocompatibility and significantly enhanced the clinical and radiographic outcomes of periodontal regeneration in one‐wall intrabony defects, and the level of clinical and radiographic improvement has been sustained for at least 2 years.^9^ Although there was no significant difference in periodontal parameters between the compared groups (with and without EMD) at any time point of assessment during the 2‐year follow‐up, no mid‐term or long‐term follow‐up outcomes have yet been documented.

Therefore, this study aimed to assess the clinical, radiographic, and patient‐reported 4‐year longitudinal outcomes of combination regenerative therapy using DPBM with adjunctive use of EMD in comparison with DPBM alone for the treatment of one‐wall intrabony defects in the molar regions.

## 2 MATERIALS AND METHODS

### 2.1 Study design

The current study was designed as a noninterventional follow‐up of participants previously enrolled in an RCT (Clinical Research Information Service, Republic of Korea Clinical Trials Registry KCT0004164), evaluating the potential advantages of adjunctive use of EMD in combination with DPBM for the treatment of one‐wall intrabony defects in the posterior regions.^9^ The study was approved by the local Institutional Review Board of Daejeon Dental Hospital, Wonkwang University (approval no. W2007/006‐001), and follow‐up measurements were performed between January 2016 and February 2021. The Consolidated Standards of Reporting (CONSORT) guidelines for clinical trials were followed.^10^

### 2.2 Participants

The inclusion criteria were as follows: (1) patients previously enrolled in an RCT and available at 4‐year follow‐up who were reexamined at 1‐year intervals and (2) fully understood the purpose of the long‐term follow‐up study and consequently signed the informed consent form. The exclusion criteria were as follows: (1) patients who were unwilling to participate in the 4‐year follow‐up examination and (2) extraction of teeth because of failure of supportive periodontal therapy. Full details of the baseline characteristics of the study population and information on the inclusion and exclusion criteria, randomization, recruitment, allocation concealment, and blinding procedures are reported in a previously published paper.^9^

### 2.3 Surgical treatment

After local anesthesia*, a full‐thickness mucoperiosteal flap was elevated minimally while sufficiently exposing the one‐wall intrabony defect. After applying the root conditioning with tetracycline solution (50 mg/mL) for 2 min, the test group was treated with a DPBM† with adjunctive use of EMD‡, and the control group was treated with a DPBM only. Flaps were sutured using a 4‐0 nonabsorbable polytetrafluoroethylene monofilament§ to achieve tension‐free primary closure. After 2 weeks, the sutures were gently removed, and further follow‐up examinations were performed.

### 2.4 Clinical and radiographic examinations

Clinical parameters, including CAL and probing pocket depth (PPD), were recorded using a periodontal probe^‖^ at baseline (before periodontal regenerative treatment), 2, 3, and 4 years postoperatively by a single board‐certified periodontist (JHL). All radiographic parameters, including defect depth and width, were evaluated using a medical imaging software^¶^ by a single calibrated examiner, not involved in the previous study. To evaluate the intra‐examiner reliability and validity, 10 periapical radiographic images were scored twice, and the intraclass correlation coefficients (ICCs) were obtained over 0.80.

### 2.5 Patient‐reported outcome measures

In this study, the oral health‐related quality of life (OHRQoL) was assessed at baseline, 2 years, and 4 years after regenerative treatment. Each participant completed the Oral Health Impact Profile‐14 (OHIP‐14), which includes seven oral health domains influencing psychosocial and physical health: functional limitation, physical pain, psychological discomfort, physical disability, psychological disability, social disability, and handicap.^11^, ^12^ A Likert scale was used for scoring (0 = never, 1 = hardly ever, 2 = occasionally, 3 = often, and 4 = very often).

### 2.6 Statistical analysis

The Shapiro–Wilk test was used to assess whether the distribution was normal, and included parameters were not normally distributed. The two‐sided Mann–Whitney U‐test was used to compare the control and test groups. Variables were expressed as mean ± standard deviation, and their 95% CIs were calculated. All statistical analyses were performed using statistical software**, and the level of statistical significance was set at *P* < 0.05.

## 3 RESULTS

**FIGURE 1:**
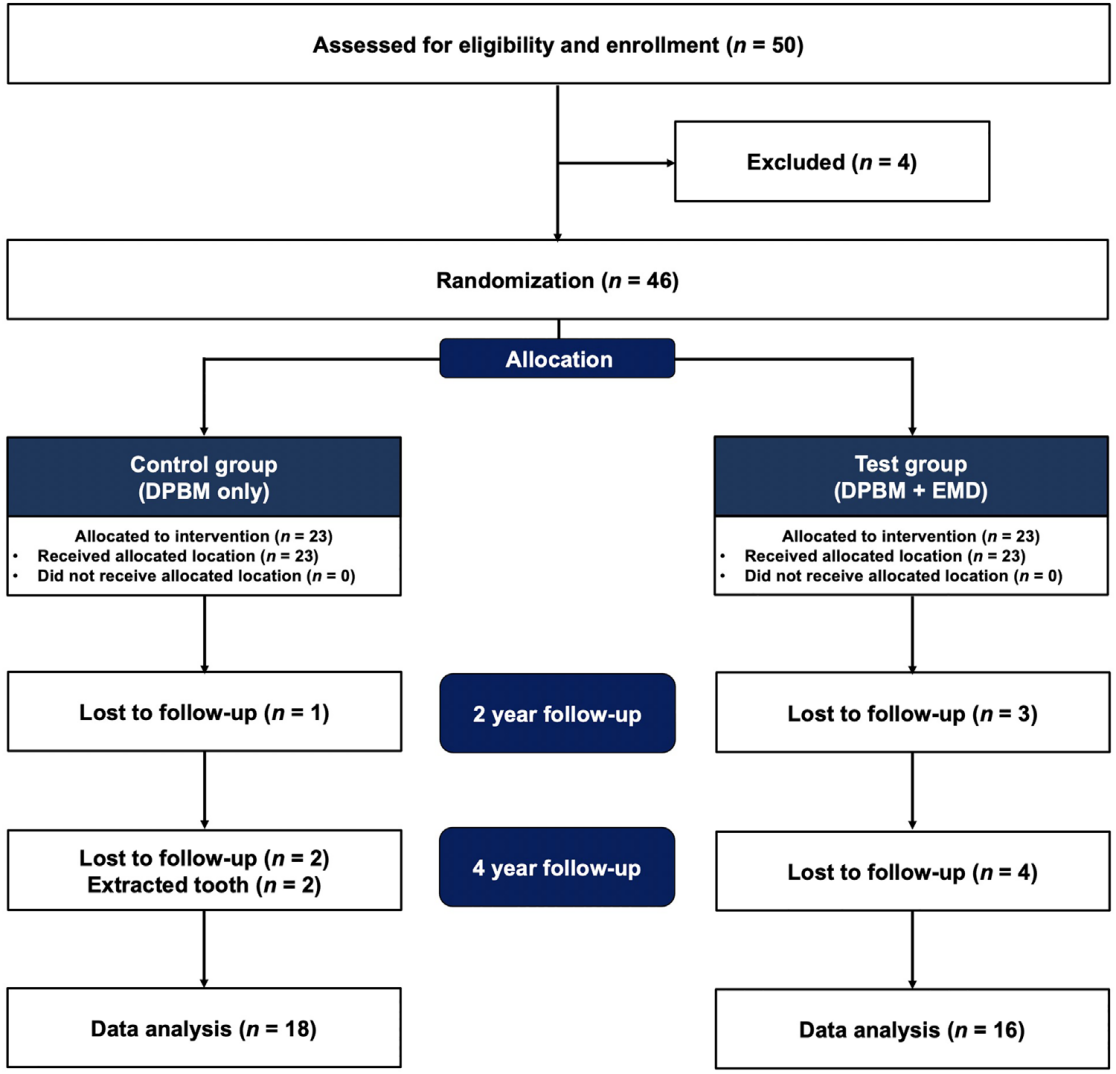
Flowchart of the participants in this study. DPBM, demineralized porcine bone matrix; EMD, enamel matrix derivative

**FIGURE 2:**
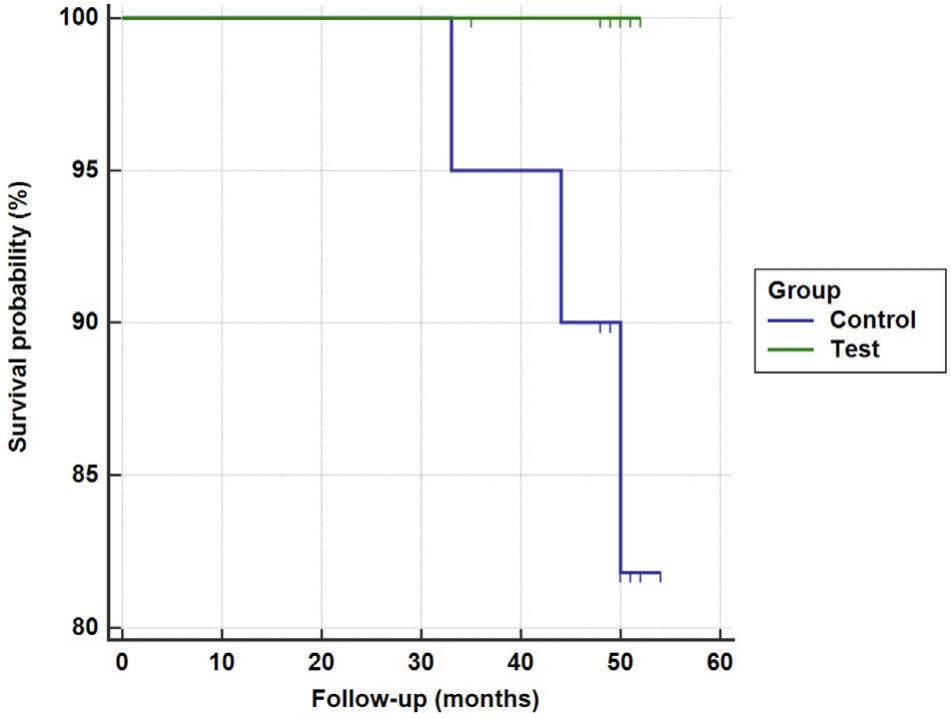
The 4‐year cumulative incidence of tooth loss. Survival probability showed no significant difference between the control and test groups (*P* = 0.136)

Of the 50 eligible participants, 34 participants were enrolled, as displayed in the flowchart of the participants in Figure 1. All participants were randomly assigned with a 1:1 allocation ratio to the control (*n* = 23; mean age 55.9 ± 12.7 years; 12 males and 11 females) and test (*n* = 23; mean age 52.3 ± 9.9 years; eight males and 15 females) groups at baseline. Of the 46 patients who enrolled in the RCT, five participants in the control group and seven participants in the test group were lost to follow‐up or lost tooth during the 4‐year period. The remaining control (*n* = 18; mean age 54.9 ± 13.1 years; 10 males and eight females) and test (*n* = 16; mean age 52.3 ± 8.8 years; six males and 10 females) groups were included in the final analysis. The Kaplan‐Meier analysis of the 4‐year cumulative incidence of tooth loss showed no significant difference between the control and test groups (*P* = 0.136) (Figure 2).

### 3.1 Clinical and radiographic outcomes

**FIGURE 3:**
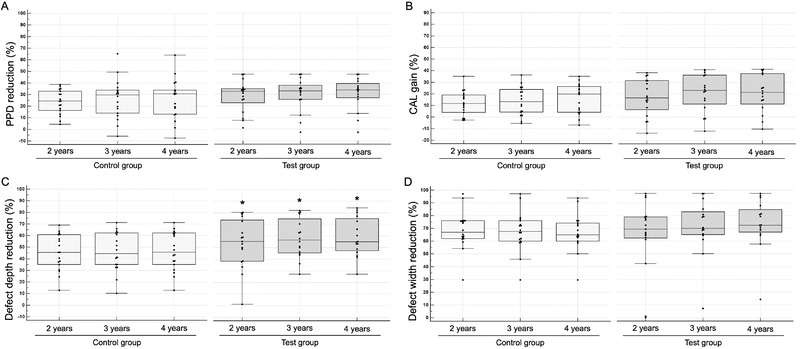
Data are presented using Box–Whisker plots showing minimum, maximum, median, and 25th and 75th percentiles. (**A**) and (**B**) show the clinical outcomes at 2, 3, and 4 years as probing pocket depth (PPD) and clinical attachment level (CAL). (**C**) and (**D**) show the radiographic outcomes at 2, 3, and 4 years as defect depth and width. Statistically significant differences between the two groups were determined (*P < 0.05)

All clinical and radiographic parameters are presented in Tables 1 and Figure 3, respectively. Two years after the regenerative treatment of 1‐wall intrabony defects, significant improvements in PPD and CAL were found in the control and test groups (*P* < 0.001). At 2 years, the control group showed a significant change in PPD from 7.3 ± 0.6 to 5.4 ± 0.8 mm (*P* < 0.001) and a significant change in CAL from 7.8 ± 0.6 to 6.7 ± 0.9 mm (*P* < 0.001), whereas the test group showed a significant change in PPD from 7.8 ± 1.0 to 5.4 ± 0.7 mm (*P* < 0.001) and a significant change in CAL from 8.5 ± 1.3 to 6.9 ± 0.8 mm (*P* < 0.001). In both groups, the level of clinical improvement observed at 2 years was sustained over a 4‐year follow‐up period. No significant differences were found between the two groups at any time point of assessment.

**TABLE 1:** Clinical and radiographic outcomes at baseline, 2 years, 3 years, and 4 years after treatment of one‐wall intrabony defects

	**Control group**							**Test group**						
	**Baseline**	**2‐year follow‐up**		**3‐year follow‐up**		**4‐year follow‐up**		**Baseline**	**2‐year follow‐up**		**3‐year follow‐up**		**4‐year follow‐up**	
**Parameters (mm)**	**Mean ± SD (95% CI)**	**Mean ± SD (95% CI)**	***P* ^a^**	**Mean ± SD (95% CI)**	***P* ^b^**	**Mean ± SD (95% CI)**	***P* ^c^**	**Mean ± SD (95% CI)**	**Mean ± SD (95% CI)**	***P* ^a^**	**Mean ± SD (95% CI)**	***P* ^b^**	**Mean ± SD (95% CI)**	***P* ^c^**
**Clinical outcomes**														
PPD	7.3 ± 0.6	5.4 ± 0.8	**<0.001**	5.3 ± 1.1	0.747	5.4 ± 1.1	0.887	7.8 ± 1.0	5.4 ± 0.7	**<0.001**	5.3 ± 0.7	0.771	5.3 ± 0.8	0.853
	(7.0‐7.5)	(5.1‐5.7)		(4.9–5.8)		(4.9–5.8)		(7.3‐8.2)	(5.1‐5.7)		(5.0‐5.6)		(4.9–5.6)	
CAL	7.8 ± 0.6	6.7 ± 0.9	**<0.001**	6.7 ± 1.0	0.907	6.6 ± 1.0	0.776	8.5 ± 1.3	6.9 ± 0.8	**<0.001**	6.6 ± 0.8	0.171	6.6 ± 0.8	0.948
	(7.6‐8.1)	(6.4‐7.1)		(6.3‐7.1)		(6.2‐7.0)		(7.9–9.1)	(6.6‐7.3)		(6.2‐6.9)		(6.2‐6.9)	
**Radiographic outcomes**														
Defect depth	4.3 ± 0.6	2.3 ± 0.5	**<0.001**	2.3 ± 0.6	0.826	2.3 ± 0.6	0.909	4.6 ± 0.8	2.1 ± 0.8	**<0.001**	1.9 ± 0.7	0.382	1.8 ± 0.7	0.875
	(4.0‐4.5)	(2.1‐2.5)		(2.0‐2.5)		(2.0‐2.5)		(4.3‐5.0)	(1.7–2.4)		(1.6–2.1)		(1.5–2.1)	
Defect width	3.3 ± 0.6	1.1 ± 0.5	**<0.001**	1.1 ± 0.4	0.133	1.1 ± 0.4	0.984	3.5 ± 0.9	1.0 ± 0.5	**<0.001**	0.9 ± 0.4	0.279	0.8 ± 0.4	0.678
	(3.0‐3.5)	(0.9–1.3)		(0.9–1.2)		(0.9–1.2)		(3.1‐3.9)	(0.8–1.2)		(0.7–1.1)		(0.6–1.0)	

Abbreviations: CAL, clinical attachment level; CI, confidence interval; PPD, probing pocket depth; SD, standard deviation.

Mean values ± SD are presented; boldface denotes statistical significance (*P* < 0.05).

*P* values for comparisons between ^a^baseline versus 2 years, ^b^2 years versus 3 years, and ^c^3 years versus 4 years, respectively.

Radiographically, significant improvements in defect depth and width were found in the control and test groups 2 years after regenerative treatment (*P* < 0.001). At 2 years, the control group showed a significant reduction in defect depth from 4.3 ± 0.6 to 2.3 ± 0.5 mm (*P* < 0.001) and a significant reduction in defect width from 3.3 ± 0.6 to 1.1 ± 0.5 mm (*P* < 0.001), whereas the test group showed a significant reduction in defect depth from 4.6 ± 0.8 to 2.1 ± 0.8 mm (*P* < 0.001) and a significant reduction in defect width from 3.5 ± 0.9 to 1.0 ± 0.5 mm (*P* < 0.001). In both groups, the level of radiographic improvement was maintained for over 4 years. In particular, for defect depth, there was a statistically significant difference between the two groups at any time point of assessment (*P* < 0.05).

### 3.2 Patient‐reported outcomes

The mean total OHIP‐14 score in both groups significantly decreased after 4 years of regenerative treatment. In particular, OHIP‐14 outcomes revealed a statistically significant reduction in the following domains (*P* < 0.05): physical pain, psychological discomfort, and physical disability. The largest improvement was observed in both groups for physical disability from baseline to 2 years; a score dropped in the control group from 1.89 ± 1.13 to 1.11 ± 0.90 (*P* = 0.029) and the test group from 1.78 ± 1.11 to 1.06 ± 0.87 (*P* = 0.038) (Table 2).

**TABLE 2:** Distribution of the OHIP‐14 questionnaire at baseline, 2 years, and 4 years after treatment of one‐wall intrabony defects

	**Control group**					**Test group**				
	**Baseline**	**2‐year follow‐up**		**4‐year follow‐up**		**Baseline**	**2‐year follow‐up**		**4‐year follow‐up**	
**OHIP score**	**Mean ± SD(95% CI)**	**Mean ± SD(95% CI)**	***P* ^a^**	**Mean ± SD(95% CI)**	***P* ^b^**	**Mean ± SD(95% CI)**	**Mean ± SD(95% CI)**	***P* ^a^**	**Mean ± SD(95% CI)**	***P* ^b^**
OHIP total score	1.21 ± 1.15	0.83 ± 0.85	**0.002**	0.80 ± 0.83	0.764	1.21 ± 1.16	0.78 ± 0.83	**0.001**	0.77 ± 0.84	0.939
	(0.75–1.71)	(0.48–1.19)		(0.45–1.15)		(0.72–1.96)	(0.44–1.11)		(0.42–1.12)	
Functional limitation	0.44 ± 0.70	0.33 ± 0.77	0.654	0.39 ± 0.61	0.811	0.50 ± 0.86	0.33 ± 0.59	0.502	0.39 ± 0.70	0.799
	(0.15–0.74)	(0.00–0.67)		(0.12–0.66)		(0.14–0.86)	(0.07–0.59)		(0.08–0.69)	
Physical pain	2.56 ± 0.86	1.89 ± 0.68	**0.014**	1.78 ± 0.81	0.658	2.28 ± 0.96	1.61 ± 0.92	**0.040**	1.72 ± 0.83	0.705
	(2.20‐2.91)	(1.59–2.19)		(1.42–2.13)		(1.88–2.68)	(1.21‐2.01)		(1.36–2.08)	
Psychological discomfort	1.67 ± 0.91	1.11 ± 0.47	**0.027**	1.00 ± 0.49	0.491	1.44 ± 0.78	0.94 ± 0.54	**0.032**	1.11 ± 0.68	0.419
	(1.29–2.05)	(0.90‐1.32)		(0.79–1.21)		(1.12–1.77)	(0.71–1.18)		(0.81–1.41)	
Physical disability	1.89 ± 1.13	1.11 ± 0.90	**0.029**	1.00 ± 0.91	0.715	1.78 ± 1.11	1.06 ± 0.87	**0.038**	0.94 ± 0.94	0.715
	(1.42–2.36)	(0.72–1.51)		(0.60‐1.40)		(1.31‐2.24)	(0.67–1.44)		(0.53–1.36)	
Psychological disability	0.50 ± 0.86	0.28 ± 0.46	0.340	0.22 ± 0.43	0.710	0.56 ± 0.92	0.33 ± 0.49	0.372	0.28 ± 0.46	0.727
	(0.14–0.86)	(0.08–0.48)		(0.03–0.41)		(0.17–0.94)	(0.12–0.55)		(0.08–0.48)	
Social disability	0.83 ± 0.92	0.61 ± 0.70	0.421	0.67 ± 0.84	0.830	0.94 ± 1.16	0.72 ± 0.75	0.500	0.61 ± 0.70	0.649
	(0.45–1.22)	(0.31–0.92)		(0.30‐1.03)		(0.46–1.43)	(0.39–1.05)		(0.31–0.92)	
Handicap	0.72 ± 0.83	0.50 ± 0.62	0.367	0.56 ± 0.62	0.789	0.94 ± 1.21	0.44 ± 0.51	0.116	0.33 ± 0.49	0.508
	(0.38–1.07)	(0.23–0.77)		(0.29–0.83)		(0.44–1.45)	(0.22–0.67)		(0.12–0.55)	

Abbreviation: OHIP‐14, Oral Health Impact Profile ‐14.

Mean values ± SD are presented; boldface denotes statistical significance (*P* < 0.05).

*P* values for comparisons between ^a^baseline versus 2 years and ^b^2 years versus 4years, respectively.

## 4 DISCUSSION

In recent years, EMD used in combination with various bone grafting materials (including autograft, allograft, xenograft, and alloplast), which is a well‐established and practical technique for significantly improved clinical and radiographic outcomes, has been particularly useful when mechanical support for large and advanced intrabony defects is deemed necessary.^4^ Specifically, adjunctive use of bone substitutes with EMD resulted in improved PPD reduction (0.40 mm, 95% CI 0.15–0.64), CAL gain (0.41 mm, 95% CI 0.13–0.69), and radiographic defect gain (0.67 mm, 95% CI 0.40–0.94) compared with EMD alone.

Given the results of our study, regardless of whether with or without adjunctive use of EMD, we found that periodontal regenerative treatment using DPBM resulted in statistically significantly improved PPD (mean 2.45 ± 1.21 mm) reduction, CAL (mean 1.58 ± 1.34 mm) gain, and defect depth (mean 2.39 ± 1.05 mm) and width (mean 2.41 ± 0.90 mm) reduction in one‐wall intrabony defects (*P* < 0.001). In addition to this, periodontal‐related OHRQoL scores also showed significant improvement from baseline to 2 year (*P* < 0.05), with no statistically significant difference between the two groups, and the scores were maintained for over 4 years. Although all enrolled participants with wide and deep one‐wall intrabony defects, only two teeth associated with severe periodontal disease were lost during the follow‐up period of 4 years after regenerative treatment, and a total survival rate of 91.7% was achieved.

These results are consistent with similar comparative RCT studies of regenerative treatment with bone substitutes combined with EMD. Zucchelli et al. reported that DBBM with EMD in deep and angular intrabony defects has the ability to significantly improve clinical (mean gain in CAL of 5.8 ± 1.1 mm) and radiographic (mean gain in defect fill of 5.3 ± 1.1 mm) outcomes achievable between baseline and 1 year.^13^ Another prospective multicenter RCT showed clinical (mean gain in CAL of 4.1 ± 3.6 mm) and radiographic (mean defect fill of 2.6 ± 1.7 mm) improvements of advanced one‐ and two‐wall intrabony defects obtained with regenerative treatment with a combination of EMD and synthetic bone graft over a period of 3 years.^14^

Few studies have addressed patient‐reported outcomes, including overall patient satisfaction and functional status, with regard to the regenerative treatment of intrabony defects.^15^, ^16^ Accumulating evidence indicates that aggressive and chronic periodontal disease has a negative effect on the general and OHRQoL of an individual, with increased impacts of significantly greater disease duration and severity.^17^, ^18^ The mean total OHIP‐14 score significantly decreased in both groups after regenerative treatment (*P* < 0.05), and OHIP results showed a sustained improvement in quality of life through the 4‐year follow‐up period. In particular, reduction of physical pain and disability after regenerative treatment may contribute to sufficient masticatory function and good occlusion. These findings were consistent with the results of a previous study that showed moderate to high OHIP levels maintained over 5 years after periodontal treatment.^19^

The variability of clinical and radiographic outcomes in periodontal regeneration is influenced by several practical factors, including surgical skill and expertise.^4^ In particular, several studies have confirmed that modified minimally invasive surgical and nonsurgical treatment modalities with papillary preservation techniques are superior to conventional open flap debridement for the treatment of intrabony defects.^20^, ^21^ In this study, because of the lack of visibility and accessibility of the surgical field in one‐wall intrabony defects compared to the two‐ or three‐wall intrabony defects, a minimally invasive approach was not actively implemented. Further consideration of the technique for evidence‐based minimally invasive periodontal regenerative surgery for one‐wall intrabony defects is needed.

Several limitations of the current study should be considered when interpreting the findings. First, all radiographic measurements were performed by a single examiner who was not involved in the previous RCT. Despite efforts to minimize measurement errors through high ICCs for both calibrations (ICC > 0.80 in the previous and current study), measurement bias was inevitable. Second, our sample size was small and had a relatively high dropout rate. Therefore, our statistical power was limited to detecting differences in clinical and radiographic outcomes. Finally, patient‐reported outcomes should be interpreted cautiously because bias owing to patients was not blinded.

## 5 CONCLUSION

Within the limitations of this study, the current findings indicate that DPBM showed favorable clinical, radiographic, and patient‐related outcomes of periodontal regeneration of one‐wall intrabony defects in a 4‐year long‐term follow‐up period. However, no additional clinical and radiographic benefits were observed with the adjunctive use of EMD.

## CONFLICT OF INTEREST

The authors declare that there are no conflicts of interest regarding this study.

## AUTHOR CONTRIBUTIONS

Conceptualization: Jae‐Hong Lee; Data curation: Jae‐Hong Lee, Seong‐Nyum Jeong; Formal analysis: Jae‐Hong Lee, Seong‐Nyum Jeong; Funding acquisition Jae‐Hong Lee; Investigation: Jae‐Hong Lee, Seong‐Nyum Jeong; Methodology: Jae‐Hong Lee, Seong‐Nyum Jeong; Project administration: Jae‐Hong Lee, Seong‐Nyum Jeong; Resources: Jae‐Hong Lee, Seong‐Nyum Jeong; Software: Jae‐Hong Lee; Supervision: Jae‐Hong Lee, Seong‐Nyum Jeong; Validation: Jae‐Hong Lee, Seong‐Nyum Jeong; Writing—original draft: Jae‐Hong Lee, Seong‐Nyum Jeong; Writing—review & editing: Jae‐Hong Lee, Seong‐Nyum Jeong.

## Footnotes

* 2% lidocaine HCl with 1:100 000 epinephrine, Yuhan, Seoul, Korea

† THE Graft 0.25g, Purgo Biologics, Seongnam, Korea

‡ Emdogain 0.3 ml, Straumann, Basel, Switzerland

§ Biotex, Purgo Biologics, Seongnam, Korea

** SAS version 9.4 software, SAS Institute, Cary, NC, USA
